# IsoFrog: a reversible jump Markov Chain Monte Carlo feature selection-based method for predicting isoform functions

**DOI:** 10.1093/bioinformatics/btad530

**Published:** 2023-08-30

**Authors:** Yiwei Liu, Changhuo Yang, Hong-Dong Li, Jianxin Wang

**Affiliations:** School of Computer Science and Engineering, Central South University, Changsha, Hunan 410083, P.R. China; Hunan Provincial Key Lab on Bioinformatics, Central South University, Changsha, Hunan 410083, P.R. China; School of Computer Science and Engineering, Central South University, Changsha, Hunan 410083, P.R. China; Hunan Provincial Key Lab on Bioinformatics, Central South University, Changsha, Hunan 410083, P.R. China; School of Computer Science and Engineering, Central South University, Changsha, Hunan 410083, P.R. China; Hunan Provincial Key Lab on Bioinformatics, Central South University, Changsha, Hunan 410083, P.R. China; School of Computer Science and Engineering, Central South University, Changsha, Hunan 410083, P.R. China; Hunan Provincial Key Lab on Bioinformatics, Central South University, Changsha, Hunan 410083, P.R. China

## Abstract

**Motivation:**

A single gene may yield several isoforms with different functions through alternative splicing. Continuous efforts are devoted to developing machine-learning methods to predict isoform functions. However, existing methods do not consider the relevance of each feature to specific functions and ignore the noise caused by the irrelevant features. In this case, we hypothesize that constructing a feature selection framework to extract the function-relevant features might help improve the model accuracy in isoform function prediction.

**Results:**

In this article, we present a feature selection-based approach named IsoFrog to predict isoform functions. First, IsoFrog adopts a reversible jump Markov Chain Monte Carlo (RJMCMC)-based feature selection framework to assess the feature importance to gene functions. Second, a sequential feature selection procedure is applied to select a subset of function-relevant features. This strategy screens the relevant features for the specific function while eliminating irrelevant ones, improving the effectiveness of the input features. Then, the selected features are input into our proposed method modified domain-invariant partial least squares, which prioritizes the most likely positive isoform for each positive MIG and utilizes diPLS for isoform function prediction. Tested on three datasets, our method achieves superior performance over six state-of-the-art methods, and the RJMCMC-based feature selection framework outperforms three classic feature selection methods. We expect this proposed methodology will promote the identification of isoform functions and further inspire the development of new methods.

**Availability and implementation:**

IsoFrog is freely available at https://github.com/genemine/IsoFrog.

## 1 Introduction

Alternative splicing (AS) is a splicing mechanism occurring in at least 90% of human genes, and it produces multiple mRNAs (isoforms) from a single gene by selectively including exons at the gene locus (David and Manley 2008, Wu *et al.* 2023). It plays an important role in gene function differentiation since the isoforms might contain distinct coding sequences, hence translated to proteins performing different biological functions (Pan *et al.* 2008, Liu *et al.* 2023). Therefore, accurate identification of isoform functions is the key to studying the functional diversity of genomes.

Despite its importance, the understanding of isoform-level biological functions is very limited so far. Also, it is impractical to make an exhaustive investigation of isoform functions through biological experiments. Therefore, computational methods leveraging the available data, e.g. RNA-seq data, for isoform function prediction are in urgent demand. Due to the lack of transcription-level annotations, many semi-supervised learning methods have been applied in isoform function prediction, such as mi-SVM (Eksi *et al.* 2013), iMILP (Li *et al.* 2014), WLRM (Luo *et al.* 2017), DeepIsoFun (Shaw *et al.* 2019), DIFFUSE (Chen *et al.* 2019), IsoFun (Yu *et al.* 2020), DisoFun (Wang *et al.* 2020), and IsoResolve (Li *et al.* 2021). These methods use the expression profiles as the primary input and Gene Ontology (GO) (Harris *et al.* 2004) terms as the function labels, and can be divided into four categories: multiple instance learning (MIL)-based methods, network propagation-based methods, matrix factorization-based methods, and domain adaptation (DA)-based methods. For example, mi-SVM, WLRM, and DIFFUSE regard isoform function prediction as a MIL problem (Maron and Lozano-Pérez 1997), in which each gene is considered as a “bag” and each isoform as an “instance.” Specifically, mi-SVM iteratively builds SVM models by relabeling the isoforms of positive genes according to their prediction scores in each iteration. DIFFUSE applies a MIL framework, which combines a Deep Neural Network for sequence feature extraction and a Conditional Random Field for isoform label assignment. Network propagation-based methods, such as iMILP and IsoFun, solve this problem by propagating the labels of genes to isoforms through their inner- and inter-relations. Based on matrix factorization, DisoFun projects the isoform expression matrix and gene-GO term relation matrix into a latent factor space, from which the relationships between isoforms and functions can be mined. IsoResolve combines Partial Least Square (PLS) regression with DA to align gene and isoform domains and then applies gene labels to isoform function prediction.

In the above methods, the same feature matrices are input for predicting different functions. However, the relevance of each feature to a function can vary greatly and the features associated with different functions can also be different. Therefore, it is of great value to integrate a feature filtering process into an isoform function prediction model, which can help screen the function-relevant features and remove the features that are irrelevant to the function.

In this article, we propose IsoFrog, a feature selection-based approach to predict isoform functions. First, we adopt an RJMCMC-based feature selection framework to calculate the feature importance to the function of interest. Second, we use a sequential feature selection (SFS) procedure to extract a subset of features that are associated with the function. In this case, the feature effectiveness is improved as the function-relevant features are selected and the irrelevant ones are filtered out. Finally, the selected features are input to our proposed method modified domain-invariant partial least squares (MdiPLS), which applies a MIL module to exploit more labeled data from positive MIGs for diPLS to predict isoform functions. IsoFrog uses RNA-seq data as the input and the GO slim terms (Shaw *et al.* 2019) as the evaluation reference. We applied IsoFrog to three datasets with 5-fold cross-validation (CV) experiments. The performance of IsoFrog was compared with IsoResolve, DisoFun, IsoFun, WLRM, iMILP, and mi-SVM. We also compared the feature selection framework in IsoFrog with three classic feature selection methods, including the least absolute shrinkage and selection operator (Lasso), F-test, and Random Forest. Next, we investigated the performance of IsoFrog on different GO term sizes and categories. Then, the contribution of each module of IsoFrog to its performance was measured with an ablation study. We also compared the selected and unselected features in their performance and the expression information contained in them. The capability of IsoFrog to identify isoform functions was then illustrated with a case study. Finally, we compared the time cost of IsoFrog and the other six state-of-the-art methods. Our results show that Isofrog can not only single out a subset of features related to the functions but also significantly improve the prediction performance over the previously reported results.

## 2 Materials and methods

### 2.1 Data collection and preprocessing

In this article, three datasets were collected from previous studies to evaluate the performance of Isofrog and other state-of-the-art methods. The summary description of the datasets is provided in Supplementary Table S1.

Dataset A (Chen *et al.* 2019) contains expression data derived from 4643 human RNA-seq experiments from the NCBI Sequence Read Archive (SRA) (Leinonen *et al.* 2011). The expressions are measured in Transcripts Per Million. The isoforms with the alignment ratio of reads lower than 0.7 are filtered out. Finally, 19 303 genes, of which 10 271 are single-isoform genes (SIGs) and 9032 are multiple-isoform genes (MIGs), 39 375 isoforms, and their expression data from 1735 experiments are retained.

Dataset B (Eksi *et al.* 2013) consists of expression data derived from 811 mouse RNA-seq experiments from the NCBI SRA database. The expressions are measured in fragments per kilobase of exon per million fragments mapped (FPKM). Experiments with few reads or low mapping rates are screened out, and isoforms not expressed (FPKM<1) in over half of the experiments are omitted. Finally, Dataset B contains data on 19 201 genes (15 974 SIGs and 3227 MIGs) and 24 274 isoforms from 365 RNA-Seq experiments.

Dataset C (Li *et al.* 2014) contains 29 datasets retrieved from human full-length isoform sequencing studies in the NCBI SRA database. Each dataset includes at least six experiments. A protein can be retained as the coefficient of variation (the ratio of standard deviation to mean) of its expression profile is 0.3, and it is significantly expressed (FPKM>10) in at least two experiments. Consequently, 19 217 genes (11 799 SIGs and 7418 MIGs), 34 340 isoforms, and their expression data from 456 SRA experiments are preserved.

Gene labels are obtained from gene functional annotations. GO slim terms (Shaw *et al.* 2019), which represent a broad range of key biological functions, are used in the experiments. We remove the GO terms that represent too specific (size < 5) or too general (size > 1000) functions and finally obtain 96 GO slim terms. Based on the newest version (11 June 2023) of the GO knowledgebase, there are no hierarchy relationships among the 96 GO slim terms.

### 2.2 Method

#### 2.2.1 Input data in both the gene and isoform domains

The input includes the expression data of genes and their isoforms. For each dataset, we denote the number of genes as *n*_g_, the number of isoforms as *n*_iso_, and the number of RNA-seq experiments as *n_f_*. An $n_{\mathrm{iso}}\times n_{f}$ matrix $X_{\mathrm{iso}}$ is used to represent the expression profiles of isoforms, where each column is regarded as a feature and stores the expression values of all the isoforms in a specific RNA-seq experiment. Similarly, an $n_{g}\times n_{f}$ matrix $X_{g}$ represents the expression data of genes.

For a given GO term, if a gene is annotated with it, then the gene is tagged with 1, otherwise with 0. The label information for genes is stored in an $n_{g}\times 1$ binary vector $y_{g}$.

#### 2.2.2 Modified domain-invariant PLS

diPLS (Nikzad-Langerodi *et al.* 2018) introduces a domain regularizer into PLS to achieve label transfers. Specifically, it projects the gene- and isoform-domain data into a latent variable (LV) space, where the two domains are aligned. Then, the gene function information can be leveraged in isoform-level prediction. Denoting the single weight vector of the LV space as **w**, we use $t_{g}$ and $t_{\mathrm{iso}}$ to represent the projections of gene and isoform feature matrices on **w,** respectively, then


(1) $$\begin{array}{c} t_{g}=X_{g}w, \end{array}$$

(2) $$\begin{array}{c} t_{\mathrm{iso}}=X_{\mathrm{iso}}w. \end{array}$$

To align the gene domain and isoform domain, **w** is calculated to minimize the difference between the distributional means and variances of $t_{g}$ and $t_{\mathrm{iso}}$. As the alignment of means has been achieved by the mean centering of $X_{g}$ and $X_{\mathrm{iso}}$, the variances are aligned by extending the PLS formula as where the first term is the PLS objective function, the second term is the domain regularizer, and *λ* is its penalty factor. Weight vector calculation follows an iterative manner, as $X_{g},\;X_{\mathrm{iso}}$, and $y_{g}$ are deflated in each iteration.


(3) $$\begin{array}{c} w=\underset{w}{\arg\min}||X_{g}-y_{g}w^{T}|{|}_{F}^{2}+\lambda |\mathrm{var}(t_{g})-\mathrm{var}(t_{\mathrm{iso}})|, \end{array}$$

Only gene labels are considered in diPLS. However, some of the isoforms can also provide label information for model training, such as the isoforms of SIGs, whose labels are directly inherited from their source genes. Here, we modify the diPLS objective function as


(4) $$\begin{array}{c} w=\underset{w}{\arg\min}||X_{g}-y_{g}w^{T}|{|}_{F}^{2}+\lambda |\mathrm{var}(t_{g})-\mathrm{var}(t_{\mathrm{iso}})|+\\ ||X_{ts}-y_{ts}w^{T}|{|}_{F}^{2}. \end{array}$$

In Equation (4), we introduce the third term as a new PLS term to include the expression data ($X_{ts}$) and labels ($y_{ts}$) of the isoforms of SIGs.

Besides SIGs, the labels of MIGs can also provide functional information for their isoforms by applying the basic idea of MIL. Specifically, the isoforms of a negative MIG are all negative, while for each positive MIG, at least one isoform is responsible for the positive annotation. Also, it is assumed that the isoforms carrying out the same function might have similar expression profiles. Following these assumptions, we propose an iterative process identifying the potentially responsible isoform for each positive MIG. First, we define $X\prime_{ts}$ as the expression data of the isoforms of SIGs and negative MIGs, and denote their labels as $y\prime_{ts}$. Then the initial weight vector $w_{0}$ is calculated with Equation (4). A reference distribution is formed by projecting all the positive SIGs onto $w_{0}$. Then, we define the projection score of an isoform as the distance between its projection and the mean of the reference distribution. For each positive MIG, the isoform with the minimum projection score is annotated as positive. We concatenate the feature matrix of these isoforms with $X\prime_{ts}$ and their labels with $y\prime_{ts}$, respectively; The steps above are looped until the set of selected isoforms is unchanged or the number of iterations exceeds 100. The schematic of the MIL process has been shown in Supplementary Fig. S1. After $X\prime_{ts}$ and $y\prime_{ts}$ have been updated, we continue to use Equation (4) to calculate the MdiPLS weight vectors.

After *n_k_* iterations of weight vector extraction (*n_k_* denotes the dimension of the LV space), we obtain an $n_{f}\times n_{k}$ weight matrix **W** in which each column represents a weight vector. Then, the regression coefficient vector **b** can be calculated as

(5) $$\begin{array}{c} b=W{\left(P^{T}W\right)}^{-1}q, \end{array}$$

where **P** is an $n_{f}\times n_{k}$ matrix and each column of **P** is a loading vector, with elements reflecting the importance of each feature. **q** is an $n_{k}\times 1$ vector storing the importance of each LV. The calculation of **P** and **q** are detailed in Supplementary Note S1. Finally, the prediction scores of isoforms can be calculated below:

(6) $$\begin{array}{c} y_{\mathrm{pred}}=X_{\mathrm{iso}}b, \end{array}$$

where $y_{\mathrm{pred}}$ stands for the predicted scores of isoforms. The higher $y_{\mathrm{pred}}$ is, the more likely the isoform is to carry out the function.

The entire algorithm procedure of MdiPLS, including the MIL process for updating $X\prime_{ts}$ and $y\prime_{ts}$, the diPLS-based LV extraction, and the calculation of regression coefficients and prediction scores, have been described in Supplementary Note S1.

#### 2.2.3 IsoFrog

**Figure 1.**
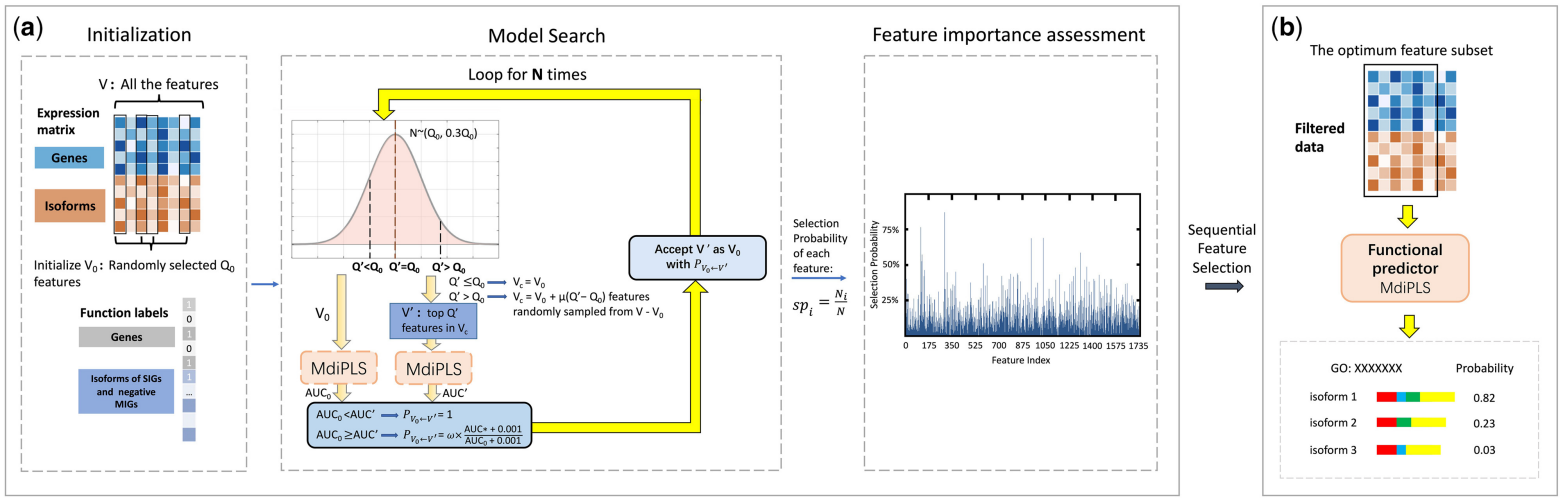
Schematic of Isofrog. (a) An RJMCMC-based feature selection framework for assessing the importance of each feature to the function of interest. (b) The predictor MdiPLS using the selected features for isoform function prediction.

The IsoFrog workflow is illustrated in Fig. 1. IsoFrog includes three steps, which will be described in detail in the following subsections.

##### Step 1. Reversible jump Markov chain Monte Carlo-based feature selection framework

Reversible jump Markov chain Monte Carlo (RJMCMC) (Green 1995) is an extension of the standard MCMC methodology. It allows fixed- and trans-dimensional model searching and simulations from the posterior distributions of model space. RJMCMC has proven to be promising for feature selection (Green 1995). However, its application is limited, since a prior distribution assigned over the models should be given in advance, and the calculation of acceptance probabilities necessitates the posterior probabilities of the model states. Here, we reference Random Frog (Li *et al.* 2012), an iterative feature selection framework that borrows the merits of RJMCMC. Based on the posterior information provided in the labeled expression data, our feature selection framework realizes flexible-dimensional moves between different models, without considering the special criteria of RJMCMC. The schematic of the feature selection framework is shown in Fig. 1a.

*Parameters and initialization:* We denote the set of all the features as **V**, and the total number of features as *p*. There are five parameters controlling the operation of our feature selection framework: (i) *N*: the total number of iterations. It should be large enough to ensure convergence and coverage of all the features in the selection process. (ii) *Q*: the number of variables selected for the first running (1≤Q≤p). It is shown in Random Frog that *Q* only has little impact at the very beginning and will not significantly influence the final result. (iii) *α*: the factor tuning the variance of the normal distribution from which the cardinality Q′ of the candidate subset V′ is sampled. (iv) *μ*: the factor that determines the range of a larger subset of **V** (compared with V′), from which the candidate features will be singled out (μ≥1). (v) *ω*: the upper bound of the probability for accepting V′ if its performance is worse than the initial variable set $V_{0}$ in the iteration. The five parameters are given in advance, and Q features are randomly selected from **V** for the initial running of the feature selection process.

*Model searching and feature importance assessment:* In each iteration, Q′ is randomly sampled from a normal distribution $N(Q_{0},\alpha Q_{0})$, where *Q*_0_ is the number of features in the initial set $V_{0}$ of the iteration ($Q_{0}=Q$ in the first running). To realize the jump purpose, we set *α* to control the standard deviation of the normal distribution. In this case, Q′ can be tuned automatically and efficiently based on *Q*_0_ since the value of Q′ is taken from [$Q_{0}-\alpha Q_{0},\;Q_{0}+\alpha Q_{0}$]. When *Q*_0_ is large, the bounds of Q′ will be extended, allowing great jumps in dimensions between models. Otherwise, the sampling range of Q′ will be limited by $\alpha Q_{0}$. This mechanism ensures that the jumps can cover all the variables in **V** while refining the model dimensionality. As Q′ is determined, in the next step, a candidate subset V′ containing exactly Q′ features is extracted (see details in Supplementary Note S2). Here, MdiPLS is used to evaluate the performance of $V_{0}$ and V′ (in terms of AUC). Specifically, we split the genes in the training dataset into 3-folds. A CV experiment is performed on these folds to obtain the AUC. Then, the acceptance probability P′ is investigated to determine whether to perform the jump from $V_{0}$ to V′. If AUC${}_{0}<$ AUC’, then P′=1. Otherwise, *ω* is set as the ceiling of P′ ($0\leq \omega \leq 1,\;P\prime =\omega \times \frac{\mathrm{AUC}\prime +0.001}{AUC_{0}+0.001}$). We randomly choose a value from [0,1] and if the value is lower than the acceptance probability, we assign $V_{0}$ as V′. The purpose of allowing a jump to a subset with poorer performance is to avoid trapping in local extremums. Meanwhile, the low ceiling of the acceptance probability guarantees the overall trend of convergence to the optimal feature subset. Following the criteria, $V_{0}$ is updated in each iteration and the step is looped for *N* times to obtain *N* models with *N* feature subsets. We examine how many times feature *i* is selected into the *N* feature subsets (*N_i_*) and calculate its selection probability as


(7) $$\begin{array}{c} sp_{i}=\frac{N_{i}}{N}. \end{array}$$

The selection probability can be regarded as a measure of feature importance, assuming that the more important a feature is, the more likely it is to be selected.

##### Step 2. Feature subset extraction

We apply an SFS procedure to search for the optimal threshold for the selection probability (see details in Supplementary Note S3). The features whose selection probabilities exceed the threshold are extracted into the final feature subset. First, we use the feature subsets corresponding to each threshold to build multiple MdiPLS models until the maximum threshold is reached. With 3-fold CVs, we obtain the AUCs of these models. Then, we select the threshold with the highest AUC and extract the corresponding features to form the final feature subset $V^{*}$ for the following prediction of isoform functions.

##### Step 3. Isoform function prediction

We perform feature filtering on $X_{g}$ and $X_{\mathrm{iso}}$ with $V^{*}$, respectively. Denoting the size of $V^{*}$ as $n_{f}^{*}$, we obtain an $n_{g}\times n_{f}^{*}$ gene expression matrix $X_{g}^{*}$ and an $n_{\mathrm{iso}}\times n_{f}^{*}$ isoform matrix $X_{\mathrm{iso}}^{*}$. We use them as the input of the MdiPLS classifier to predict isoform functions.

## 3 Performance evaluation

### 3.1 Comparison of IsoFrog with state-of-the-art methods

We compare the performance of IsoFrog on Dataset A/B/C with six state-of-the-art methods (mi-SVM, iMILP, WLRM, IsoFun, DisoFun, and IsoResolve), which also use expression data as input. All the input parameters for the methods were set as suggested by the authors or optimized in the suggested ranges. Specifically, we partitioned the dataset into 5-folds. Genes from the same homologous group and their isoforms were divided into the same fold for avoiding information leakage. The homologous genes are obtained from the Duplicated Genes Database (Ouedraogo *et al.* 2012). The 5-fold CV was performed on each of the 96 GO slim terms. The values of the parameters for IsoFrog were set as recommended in Random Frog, given in Supplementary Table S2.

The means of AUCs and AURPCs for all the GO slim terms are used to evaluate the model performance. Also, we use the *t*-test to compare the performance of the methods. The 5-fold CV results and *P*-values are shown in Table 1. Since comparisons are made between IsoFrog and the other methods, only the compared methods are annotated with *P*-values. It shows that our method performs better than the six state-of-the-art methods across all the datasets. The AUCs obtained with IsoFrog on Datasets A, B, and C are 0.769, 0.834, and 0.771, respectively, significantly higher (*P*-value <.05) than those with the other compared methods. For Dataset A, the improvements of IsoFrog over WLRM, mi-SVM, DisoFun, IsoFun, iMILP, and IsoResolve are 647.2%, 236.3%, 47.0%, 36.6%, 26.3%, and 13.0%, respectively (against the baseline of 0.5). For Dataset B, the improvements are 735.0%, 224.3%, 160.9%, 92.0%, 89.8%, and 12.8%, respectively, and 222.6%, 1078.3%, 57.6%, 47.3%, 25.5%, and 17.3% for Dataset C. In terms of AUPRC, our method also outperforms the other methods. Taking Dataset A as an example, our method achieves improvements of 395.2%, 79.3%, 67.7%, 40.5%, 23.1%, and 14.9% compared with WLRM, mi-SVM, DisoFun, IsoFun, iMILP, and IsoResolve, respectively (against the baseline of 0.1). These significant improvements in performance over the existing methods on several human and mouse datasets demonstrate the effectiveness of IsoFrog in isoform function prediction. Specifically, IsoFrog outperforms the other methods, including the MIL-based methods (mi-SVM and WLRM), network propagation-based methods (IsoFun and iMILP), and matrix factorization-based methods (DisoFun), by a large margin. These methods take the same set of features as input when predicting different functions, disregarding the importance of features to each function. In contrast, the feature selection framework of IsoFrog can help screen the function-relevant features, which is the main cause for the improvement of IsoFrog over the other methods; besides, the performance superiority of IsoFrog over IsoResolve can further prove this assumption, since IsoFrog is proposed based on diPLS by adding the feature selection framework to it.

**Table 1.** The CV performance of IsoFrog and state-of-the-art methods in terms of the mean AUC and AUPRC for the 96 GO slim terms.^a^

Method	Dataset A		Dataset B		Dataset C	
	AUC	AUPRC	AUC	AUPRC	AUC	AUPRC
IsoFrog	**0.769**	**0.308**	**0.834**	**0.436**	**0.771**	**0.336**
IsoResolve	0.738 (1.0e-05)	0.281 (1.6e-04)	0.796 (2.1e-07)	0.375 (3.6e-05)	0.731 (8.9e-08)	0.292 (8.1e-05)
DisoFun	0.683 (8.8e-45)	0.224 (5.9e-24)	0.628 (3.9e-64)	0.238 (7.3e-44)	0.672 (9.2e-61)	0.215 (5.5e-36)
IsoFun	0.697 (1.2e-30)	0.248 (6.5e-10)	0.674 (1.8e-45)	0.282 (1.3e-24)	0.684 (3.8e-44)	0.230 (1.5e-29)
WLRM	0.536(1.6e-212)	0.142 (1.2e-88)	0.540 (1.9e-189)	0.163 (2.4e-76)	0.584 (2.3e-93)	0.209 (6.7e-44)
iMILP	0.713 (3.6e-22)	0.269 (1.0e-05)	0.676 (6.8e-45)	0.288 (1.4e-23)	0.716 (1.6e-22)	0.284 (4.1e-07)
mi-SVM	0.580 (2.2e-166)	0.216 (1.7e-27)	0.603 (1.7e-77)	0.204 (2.0e-57)	0.523 (1.7e-224)	0.147 (3.9e-79)

a Values in the parentheses are the *P*-values obtained from the *t*-test. Bold values indicate the maximal AUC or AUPRC among different methods.

**Figure 2.**
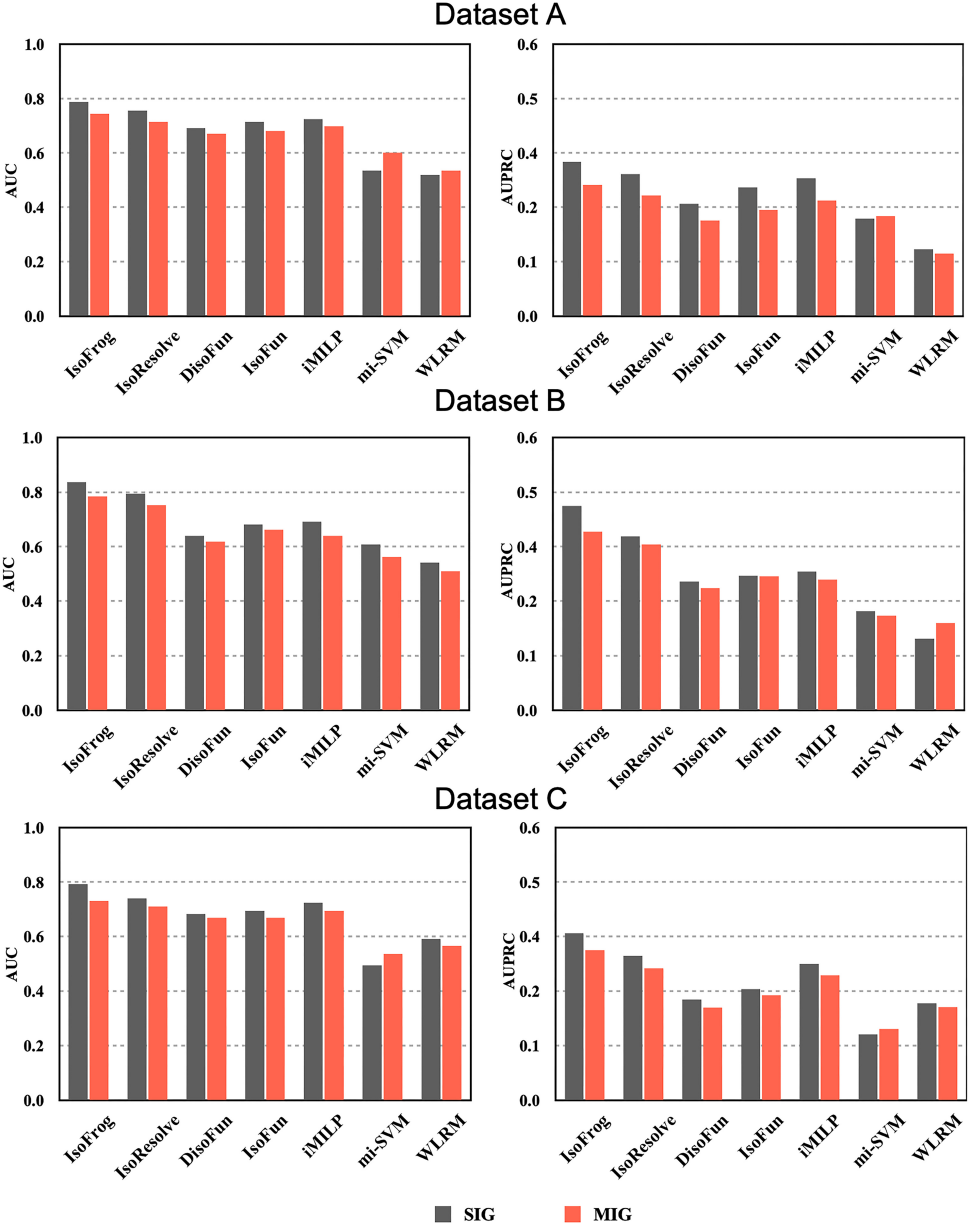
Comparison between the performance of IsoFrog and the state-of-the-art methods on SIGs and MIGs.

Model evaluation and method comparison are also performed on SIGs and MIGs separately. For SIGs, the mean AUCs of IsoFrog, IsoResolve, DisoFun, IsoFun, iMILP, mi-SVM, and WLRM on Dataset A are 0.787, 0.755, 0.691, 0.715, 0.724, 0.534, and 0.518, respectively. The AUPRCs are 0.341, 0.313, 0.247, 0.284, 0.304, 0.216, and 0.147, respectively (Fig. 2). We can see that on the three datasets, IsoFrog achieves the best performance among the seven methods, suggesting its promising ability for isoform-level function identification. As for MIGs, since there were no available isoform-level annotations, we took the maximum prediction score among the isoforms of each MIG to check its consistency with the gene annotations (Eksi *et al.* 2013, Shaw *et al.* 2019). As shown in Fig. 2, IsoFrog also shows performance superiority on MIGs over the other methods through all the datasets.

### 3.2 Comparison of the IsoFrog feature selection framework with other feature selection methods

We compare the feature selection framework in IsoFrog with three classic feature selection methods, including Lasso (Tibshirani 1996), F-test (St»hle and Wold 1989), and Random Forest (Breiman 2001). Lasso was implemented in Scikit-Learn and the magnitude of the coefficients was regarded as the importance of the corresponding features. Implementation of the ANOVA F-test was provided in the Scikit-Learn Machine Learning library, and the results were used to measure the usefulness of the features to the model. Random Forest was implemented in the rf mode in LightGBM to calculate the importance of features.

Experiments were performed on Dataset A/B/C with GO slim terms. An SFS procedure that is similar to the one in IsoFrog was applied to extract the final feature subset. MdiPLS was used as the predictor to test the performance of the features selected by IsoFrog, Lasso, F-test, and Random Forest, respectively. Also, we use the *t*-test to compare the performance of the methods. As shown in Table 2, we can see that IsoFrog achieves performance superior to all the others across the two metrics and three datasets. More specifically, IsoFrog improves the AUC and AUPRC of Random Forest by 3.9% and 8.9%, respectively, on Dataset A, 2.5% and 7.7% on Dataset B, and 3.4% and 5.4% on Dataset C. Compared with Lasso, our feature selection module achieves improvements of 5.1% in AUC and 6.1% in AUPRC on Dataset A, 4.0% and 10.2% on Dataset B, and 4.6% and 9.3% on Dataset C. Compared with F-test, IsoFrog shows an increase of 3.1% in AUC and 7.8% in AUPRC on Dataset A, 3.4% and 10.9% on Dataset B, and 5.4% and 8.3% on Dataset C. These results demonstrate the stronger power of IsoFrog in screening the function-relevant features than the classic methods. Besides, feature selection methods improve the performance of MdiPLS, supporting our initial assumption that a feature selection process can bring an improvement in model accuracy in isoform function prediction.

**Table 2.** Performance comparison of different feature selection methods with MdiPLS used as the modeling method.^a^

Method	Dataset A		Dataset B		Dataset C	
	AUC	AUPRC	AUC	AUPRC	AUC	AUPRC
All features + MdiPLS	0.750 (3.1e-04)	0.289 (2.4e-04)	0.813 (7.3e-05)	0.389 (9.7e-05)	0.744 (6.9e-06)	0.309 (7.3e-04)
F-test + MdiPLS	0.761 (6.3e-03)	0.293 (1.3e-03)	0.823 (4.0e-04)	0.403 (6.7e-04)	0.757 (1.9e-04)	0.318 (3.3e-03)
Lasso + MdiPLS	0.756 (7.2e-04)	0.296 (2.4e-03)	0.821 (2.9e-04)	0.405 (7.1e-04)	0.759 (7.6e-04)	0.316(9.7e-04)
Random Forest + MdiPLS	0.759 (1.6e-03)	0.291 (5.5e-04)	0.826 (2.6e-03)	0.412 (3.5e-03)	0.762 (9.2e-04)	0.324 (8.3e-03)
IsoFrog	**0.769**	**0.308**	**0.834**	**0.436**	**0.771**	**0.336**

a Values in the parentheses are *P*-values obtained from the *t*-test. Bold values indicate the maximal AUC or AUPRC among different methods.

### 3.3 Performances on GO terms of different sizes and functional categories

The size and category of GO terms have an impact on the performance of isoform function prediction (Eksi *et al.* 2013, Shaw *et al.* 2019, Li *et al.* 2021). GO term size refers to the number of genes annotated to the GO term, and category refers to the three main branches of GO terms, including biological process (BP), cellular component (CC), and molecular function (MF). We conducted experiments on Dataset A to investigate how the performance of IsoFrog would be influenced by the size and category of GO terms.

**Figure 3.**
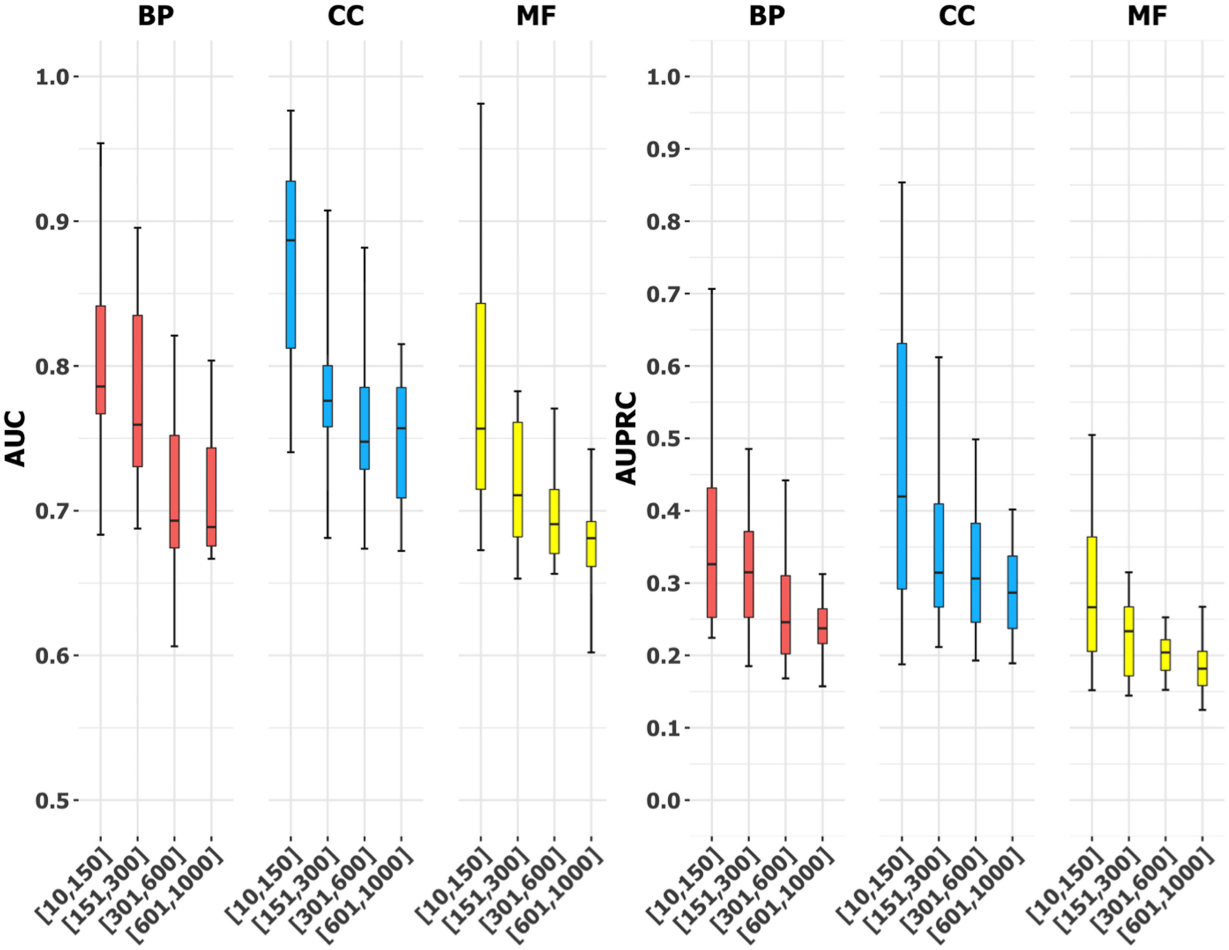
Prediction performance of IsoFrog on GO terms with different sizes in the BP, CC, and MF categories (based on Dataset A and 180 GO terms).

We divided all the GO terms into 12 groups based on their sizes and categories. First, we divided GO terms into three categories (BP, CC, and MF). Then, the terms in each category were divided into four subgroups ([10, 150], [151, 300], [301, 600], and [601, 1000]) according to their sizes. From the total of 4272 GO terms, we randomly selected 84 terms with the size of 10–1000 and combined them with the 96 GO slim terms, such that the size of each group is the same. Specifically, for the 180 GO terms, each of the 12 groups contains exactly 15 GO terms. For each GO term, both AUC and AUPRC are used as the performance metrics. The medians of AUCs for BP, CC, and MF terms are 0.734, 0.779, and 0.724, respectively. As for AUPRC, to ensure that the AUPRCs of different GO terms are comparable, we adjusted the baselines for all the GO terms to 0.1, following the strategy presented in Shaw *et al.* (2019). The median AUPRCs for the three categories are 0.285, 0.358, and 0.239, respectively. The experiment result shows the robustness of IsoFrog on the three main branches of GO terms. As shown in Fig. 3, the performance of IsoFrog degrades as GO term size increases, which is also observed in previous studies (Shaw *et al.* 2019). A potential reason is that GO terms with larger sizes represent more heterogeneous functions and contain more noise, being harder for models to predict.

### 3.4 Analysis of the effects of model components

To investigate the contribution of each module of IsoFrog to its performance, we applied an ablation study by removing the modules one by one and observing how the model performance would be affected. We introduce four variants: (i) diPLS, (ii) MdiPLS, (iii) diPLS-feat (integrating diPLS and the feature selection framework in IsoFrog), and (iv) IsoFrog. We applied the four variants to Dataset A. Following the same experimental configuration, we obtain the performance of these variants and list them in Supplementary Table S3.

**Figure 4.**
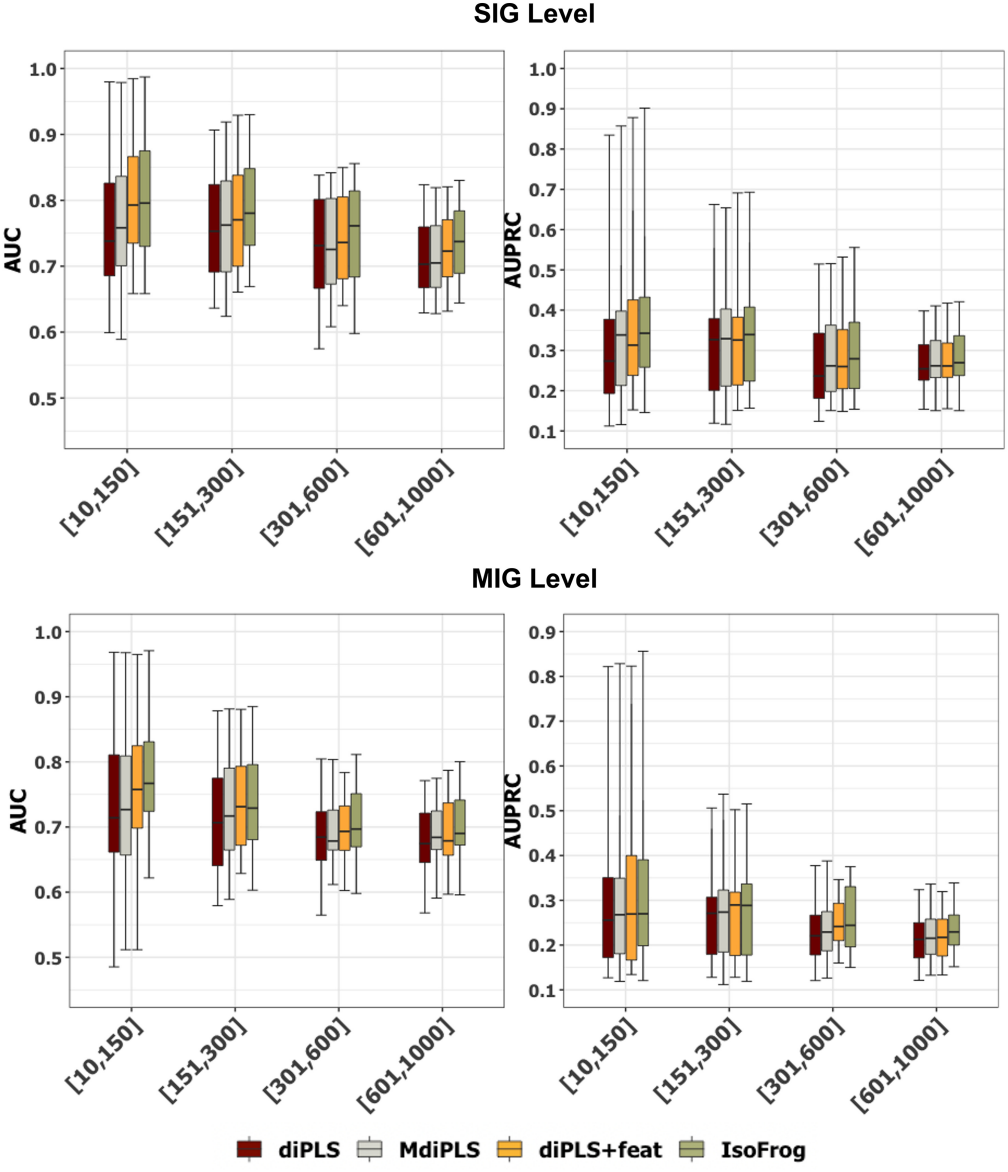
Comparison of prediction performance between diPLS, MdiPLS, diPLS-feat, and IsoFrog on different GO term sizes.

In Fig. 4, we observe that Isofrog achieves the best performance (AUC and AUPRC) among the four variants under the circumstances of different GO term sizes. For example, as GO term sizes are in [151–300], the medians of the SIG-level AUCs are 0.780, 0.770, 0.762, and 0.753 for IsoFrog, diPLS-feat, MdiPLS, and diPLS, respectively. This confirms the necessity of the feature selection module to screen the function-relevant features and the MIL module to include and update the isoform-level annotations. We obtain similar observations in the ablation experiments on the different categories of GO terms (Supplementary Fig. S2). From the ablation study, we conclude that both the feature selection module and the MIL process contribute to the improved performance of our method.

### 3.5 Comparison of the selected and unselected features by IsoFrog

**Figure 5.**
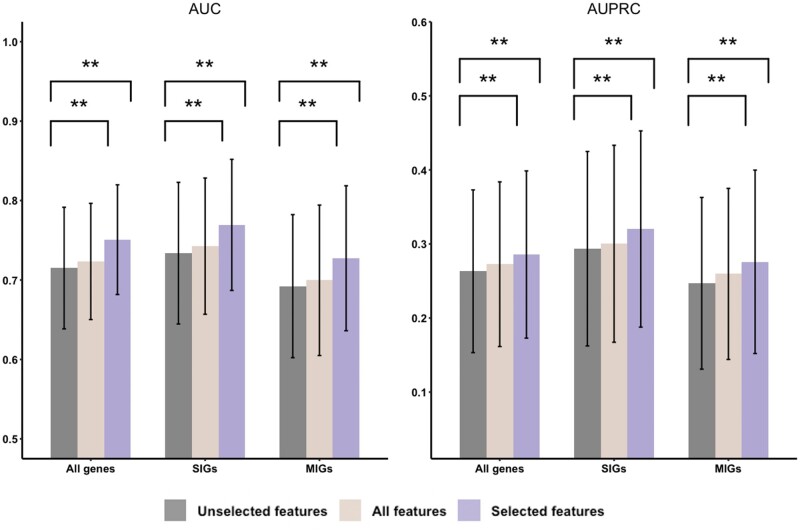
Performance comparison of MdiPLS using unselected features, all features, and selected features.

We compared the selected and unselected features to investigate whether the selected features carry information more relevant to the functions than the unselected ones. First, the prediction performance of the selected and unselected features was compared based on Dataset A. For each of the 96 GO slim terms, we defined the subset of the selected features as Set A, the unselected features as Set B, and the set of all features as Set C. Sets A, B, and C were input into MdiPLS, respectively. As shown in Fig. 5, Set A (the selected features) achieves the best performance among the three feature sets. Meanwhile, the performance of Set B (the unselected features) shows noticeable drops compared with the two others. Specifically, the AUCs and AUPRCs of Set B, whose medians are 0.721 and 0.241, are significantly lower than the ones yielded by Set C (0.726 and 0.249) and Set A (0.745 and 0.263). The result suggests that the expression information in the selected features is significantly associated with the function, while the unselected features are much less relevant to the function.

**Figure 6.**
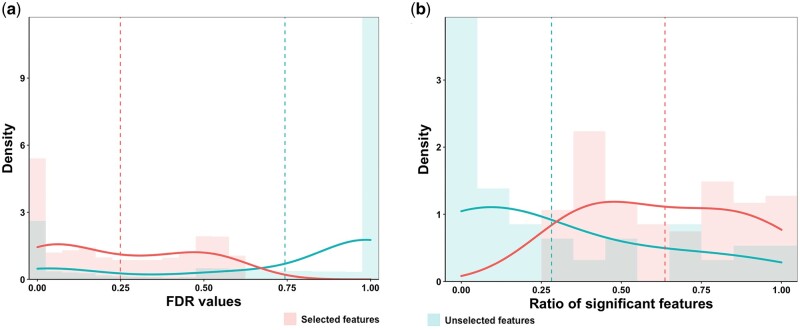
Comparison of the results of the *t*-test on selected features and unselected features. The *t*-test is applied to investigate if the expressions of the positives and negatives in these features are significantly different. (a) Histograms of the FDRs for the selected features and the unselected features of the 96 GO terms; (b) histograms of the proportions of the significant features (FDR<0.05) in the selected and unselected feature subsets for all the 96 GO terms.

Second, for each GO term, we split the 19 303 genes in Dataset A into positives and negatives, in which the former represents the genes annotated to the GO term, and the latter represents the unannotated genes. We applied the *t*-test to each feature to investigate if the positives and negatives are differentially expressed in this feature. Specifically, we extracted the expression values of the positives and negatives separately from the feature and calculated the *P*-value between the two sets of expressions. The smaller the *P*-value is, the more functional information the feature may carry. The *P*-values were FDR-adjusted to reduce false positives in the multiple-hypothesis testing. Figure 6a shows two histograms of FDRs, one for the selected features of all the GO slim terms and one for the unselected features. We can see that the selected features have FDR values significantly smaller than those of the unselected ones, implicating their stronger relevance to the functions of interest. As shown in Fig. 6b, the percentages of the significant features (FDR<0.05) in the selected subsets are significantly higher than in the unselected subsets. From the results, we conclude that compared with the unselected feature subsets, the selected subsets contain more informative features for the corresponding functions.

## 4 Case study

We further tested the performance of IsoFrog on a dataset of genes with annotated isoform functions described in the work (Shaw *et al.* 2019). Based on an exhaustive literature search, the authors extracted six genes [ACE (Corradi *et al.* 2006), ACMSD (Pucci *et al.* 2007), GCH1 (Auerbach *et al.* 2000), ADK (Cui *et al.* 2009), AIFM1 (Delettre *et al.* 2006), and PPP1R8 (Chang *et al.* 1999)] with their isoform-level annotations to “positive regulation of apoptotic process” (GO:0043065) and “metal ion binding” (GO:0046872). The isoforms of the genes can be used as a good benchmark to validate the capability of models to accurately assign gene labels to isoforms. Therefore, we tested whether the two gene functions could be differentiated at the isoform level by IsoFrog and the compared methods mentioned in Section 3.1. Specifically, we apply the softmax function to the prediction scores of isoforms to obtain the probability of being positive. An isoform would be regarded as carrying out a function if the probability is higher than 0.5. Otherwise, the isoform would be predicted to be negative. Table 3 shows the experimental results. Positive and negative results are represented as 1 and 0 respectively. As we can see from the results, the prediction performance varies greatly in different methods. For both of the GO terms, IsoFrog can successfully predict the labels of 6 out of the 7 isoforms (85.7% accuracy). As a comparison, iMILP and WLRM achieve an accuracy of 50%, mi-SVM and IsoFun achieve 57.1%, and IsoResolve and DisoFun achieve 64.3%. These results indicate that our method is more accurate in recognizing isoform functions than the other methods.

**Table 3.** Literature support for 14 isoforms of six genes on two GO terms.

GO term	Gene	Isoform	Literature	Prediction methods						
				IsoFrog	IsoResolve	DisoFun	IsoFun	WLRM	mi-SVM	iMILP
GO:0046872	ACE	P12821-1	1	1	1	1	1	1	1	1
		P12821-3	1	1	1	1	1	0	1	1
	ACMSD	Q8TDX5-1	1	1	0	1	1	1	1	0
		Q8TDX5-2	0	0	1	0	1	1	1	1
	GCH1	P30793-1	1	1	1	0	1	0	0	1
		P30793-2	0	0	0	0	1	0	1	0
		P30793-4	0	1	1	0	1	1	1	0
GO:0005634	ADK	P55263-1	1	1	1	0	0	1	1	0
		P55263-2	0	0	0	0	0	1	1	1
	AIFM1	Q95831-1	1	1	1	1	0	1	1	1
		Q95831-3	0	0	1	1	0	1	1	1
		Q95831-4	0	1	0	0	0	1	0	1
	PPP1R8	Q12972-1	1	1	1	0	1	1	1	1
		Q12972-3	0	0	0	0	1	0	0	1
Accuracy				85.7%	64.3%	64.3%	57.1%	50%	57.1%	50%
Recall				100%	85.7%	57.1%	71.4%	71.4%	85.7%	71.4%

## 5 Conclusion

In this article, we propose IsoFrog, a feature selection-based method for isoform function prediction. The contribution of IsoFrog is 2-folds. First, IsoFrog introduces an RJMCMC-based feature selection framework to assess feature importance for each function and select function-relevant features via SFS. Existing methods do not consider the features’ relevance to functions and apply the same set of features for predicting different functions. In contrast, the RJMCMC framework selects relevant features for each function, which can enhance the performance of IsoFrog. Second, we propose MdiPLS to build models for predicting isoform functions. In detail, MdiPLS first employs a MIL process to prioritize the most likely positive isoform for each positive MIG and then incorporate them to build diPLS models. This process enhances the capability of diPLS to exploit more labeled training data. We applied IsoFrog on three datasets with 5-fold CV experiments. The result shows that IsoFrog achieves superior performance over the existing methods including IsoResolve, DisoFun, IsoFun, WLRM, iMILP, and mi-SVM. Also, we compared the feature selection framework in IsoFrog with Lasso, F-test, and Random Forest. IsoFrog outperforms all the other feature selection methods on the three datasets. In the experiments, the performance of IsoFrog is robust on different GO term categories, while the performance reduces as the GO term size increases. One possible reason is that the larger-size terms might annotate genes that are more heterogeneous, resulting in difficulty in isoform function prediction. In the ablation experiment, IsoFrog achieves better performance than any other module combinations. Next, we validated that the selected features are more informative for their corresponding functions than the unselected ones. Finally, we illustrated the capability of our method for isoform function identification with a case study. Our results show that Isofrog can not only single out a subset of function-relevant features but also lead to significant performance improvements over the previously reported results.

We also analyzed the time cost of IsoFrog. Specifically, we calculated the time cost of CV using the seven methods on the same machine with CPU 32 Intel(R) Xeon(R) Gold 5218 CPU@2.30 GHz. The experiments were performed on the 96 GO slim terms with Dataset A. The running time for IsoFrog, IsoFun, iMILP, DisoFun, WLRM, mi-SVM, and IsoResolve is 271.45, 7.54, 2.6, 656.15, 63.33, 65.55, and 2.16 min, respectively. Among the seven methods, IsoFrog is faster than DisoFun but slower than the other methods. The reason is that IsoFrog takes 2000 iterations for calculating feature importance (*N* = 2000).

Our method can be further improved in several ways, including integrating other types of genomic data, as heterogeneous data may complement each other and thus provide additional information related to isoform functions. An important problem to consider is how to design a method that can effectively integrate different types of data, such as sequences, conserved domains, protein–protein interactions, GO hierarchy, etc. The tissue-specific data and disease-associated data may also bring new perspectives to isoform function prediction (Chen *et al.* 2021). We expect that more powerful methods for isoform function prediction will be developed in the future and that the isoform-level function annotations will advance our understanding of biological processes and disease pathways.

## Supplementary Material

btad530_Supplementary_Data

## Data Availability

The data underlying this article are publicly available with their sources described in the article.
