# Supplementary material for: IsoFrog: a reversible jump Markov Chain Monte Carlo feature selection-based method for predicting isoform functions
Source: Bioinformatics. 2023 Aug 30;39(9):btad530. doi: 10.1093/bioinformatics/btad530 (PMC10491952; doi:10.1093/bioinformatics/btad530)
Supplement: btad530_Supplementary_Data [file btad530_supplementary_data.pdf]

Supplementary materials for

IsoFrog: a Reversible Jump Monte Carlo Markov Chain feature selection-based method for predicting isoform functions

List of supplementary content:

**Supplementary Note S1**	The entire algorithm procedure of MdiPLS.
**Supplementary Note S2**	The procedure of model searching in the feature selection framework of IsoFrog.
**Supplementary Note S3**	The procedure of Sequential Feature Selection.
**Supplementary Figure S1**	Schematic illustration of the MIL process in MdiPLS.
**Supplementary Figure S2**	Comparison of prediction performance between diPLS, MdiPLS, diPLS-feat, and IsoFrog on different GO term categories.
**Supplementary Table S1**	Summary description of the datasets used in our study.
**Supplementary Table S2**	Parameters of IsoFrog used in the experiments.
**Supplementary Table S3**	Results of the ablation experiment on Dataset A.

Supplementary Note S1. The entire algorithm procedure of MdiPLS [1, 2].

Updating $\boldsymbol{X'}_{ts}$ and $\boldsymbol{y'}_{ts}$ (Supplementary Figure S1). As described in the main text, The formula that computes $\boldsymbol{w}$ can be written as below:

$$\boldsymbol{w} = \underset{\boldsymbol{w}}{argmin}\|\boldsymbol{X}_g - \boldsymbol{y}_g\boldsymbol{w}^T\|_F^2 + \lambda\left|\frac{1}{n_g-1}\boldsymbol{w}^T\boldsymbol{X}_g^T\boldsymbol{X}_g\boldsymbol{w} - \frac{1}{n_{iso}-1}\boldsymbol{w}^T\boldsymbol{X}_{iso}^T\boldsymbol{X}_{iso}\boldsymbol{w}\right| + \|\boldsymbol{X'}_{ts} - \boldsymbol{y'}_{ts}\boldsymbol{w}^T\|_F^2 \quad (1)$$

Then the MIL process for updating $\boldsymbol{X'}_{ts}$ and $\boldsymbol{y'}_{ts}$ is as follows:

(1) The first weight vector $\boldsymbol{w}_0$ is calculated with Equation (1), where $\boldsymbol{X'}_{ts}$ is the isoform expression data of SIGs as well as negative MIGs, and $\boldsymbol{y'}_{ts}$ stores the isoform labels of the above genes.

(2) Project the expression data of all the positive SIGs onto $\boldsymbol{w}_0$ to form a reference distribution.

(3) Define the projection score of an isoform as the distance between the projection of its data onto $\boldsymbol{w}_0$ and the mean of the reference distribution. Given a positive MIG, its isoform with the minimum projection score will be annotated as positive, as it is assumed that isoforms carrying out the same function might have similar expression profiles.

(4) Concatenate the expression data of the selected isoforms for all the positive MIGs with $\boldsymbol{X'}_{ts}$ and their labels with $\boldsymbol{y'}_{ts}$, respectively.

(5) Recalculate $\boldsymbol{w}_0$ with Equation (1).

(6) Loop steps (2)-(5) until the set of selected isoforms is unchanged or the number of iterations exceeds 100.

Computing $\boldsymbol{W}$ and $\boldsymbol{P}$. After $\boldsymbol{X'}_{ts}$ and $\boldsymbol{y'}_{ts}$ have been updated, we obtain the formula for calculating the MdiPLS weight vectors as follows:

$$\boldsymbol{w}_{iso} = \underset{\boldsymbol{w}_{iso}}{argmin}\|\boldsymbol{X} - \boldsymbol{y}\boldsymbol{w}^T\|_F^2 + \lambda\left|\frac{1}{n_g-1}\boldsymbol{w}^T\boldsymbol{X}_g^T\boldsymbol{X}_g\boldsymbol{w} - \frac{1}{n_{iso}-1}\boldsymbol{w}^T\boldsymbol{X}_{iso}^T\boldsymbol{X}_{iso}\boldsymbol{w}\right|, \quad (2)$$

where $\boldsymbol{X} = \begin{bmatrix} \boldsymbol{X}_g \\ \boldsymbol{X'}_{ts} \end{bmatrix}$ and $\boldsymbol{y} = \begin{bmatrix} \boldsymbol{y}_g \\ \boldsymbol{y'}_{ts} \end{bmatrix}$. $\boldsymbol{w}_{iso}$ represents the first column vector of $\boldsymbol{W}$ (corresponding to the first dimension of the LV space). It is normalized as follows:

$$\boldsymbol{w}_{iso} = \frac{\boldsymbol{w}_{iso}}{\|\boldsymbol{w}_{iso}\|}, \tag{3}$$

where $\|\cdot\|$ indicates the length of $\boldsymbol{w}_{iso}$.

We use $\boldsymbol{t}_g$, $\boldsymbol{t}_{iso}$, and $\boldsymbol{t}$ to denote the projections of $\boldsymbol{X}_g$, $\boldsymbol{X}_{iso}$, and $\boldsymbol{X}$ on $\boldsymbol{w}_{iso}$, respectively. $\boldsymbol{t}_g$, $\boldsymbol{t}_{iso}$, and $\boldsymbol{t}$ can be calculated with the formulas below:

$$\boldsymbol{t}_g = \boldsymbol{X}_g \boldsymbol{w}_{iso}, \tag{4}$$

$$\boldsymbol{t}_{iso} = \boldsymbol{X}_{iso} \boldsymbol{w}_{iso}, \tag{5}$$

$$\boldsymbol{t} = \boldsymbol{X} \boldsymbol{w}_{iso}. \tag{6}$$

For each of the three projection vectors above, the corresponding loading vectors, denoted by $\boldsymbol{p}_g$, $\boldsymbol{p}_{iso}$, and $\boldsymbol{p}$, respectively, can be calculated as follows:

$$\boldsymbol{p}_g^T = \left(\boldsymbol{t}_g^T \boldsymbol{t}_g\right)^{-1} \boldsymbol{t}_g^T \boldsymbol{X}_g, \tag{7}$$

$$\boldsymbol{p}_{iso}^T = (\boldsymbol{t}_{iso}^T \boldsymbol{t}_{iso})^{-1} \boldsymbol{t}_{iso}^T \boldsymbol{X}_{iso}, \tag{8}$$

$$\boldsymbol{p}^T = (\boldsymbol{t}^T \boldsymbol{t})^{-1} \boldsymbol{t}^T \boldsymbol{X}. \tag{9}$$

Similarly, the loadings for $\boldsymbol{y}$ and $\boldsymbol{y}_g$ can be calculated as:

$$q_g = \left(\boldsymbol{t}_g^T \boldsymbol{t}_g\right)^{-1} \boldsymbol{t}_g^T \boldsymbol{y}_g, \tag{10}$$

$$q = (\boldsymbol{t}^T \boldsymbol{t})^{-1} \boldsymbol{t}^T \boldsymbol{y}. \tag{11}$$

$\boldsymbol{p}$ calculated above represents the first column vector of $\boldsymbol{P}$. Similarly, q represents the first element of $\boldsymbol{q}$.

Then, $\boldsymbol{X}_g$, $\boldsymbol{X}_{iso}$, and $\boldsymbol{X}$ are deflated by removing the contribution of the first latent variable as follows:

$$\boldsymbol{X}_g = \boldsymbol{X}_g - \boldsymbol{t}_g\boldsymbol{p}_g^T, \tag{12}$$

$$\boldsymbol{X}_{iso} = \boldsymbol{X}_{iso} - \boldsymbol{t}_{iso}\boldsymbol{p}_{iso}^T, \tag{13}$$

$$\boldsymbol{X} = \boldsymbol{X} - \boldsymbol{t}\boldsymbol{p}^T. \tag{14}$$

Similarly, $\boldsymbol{y}_g$ and $\boldsymbol{y}$ are deflated as follows:

$$\boldsymbol{y}_g = \boldsymbol{y}_g - q_g\boldsymbol{t}_g, \tag{15}$$

$$\boldsymbol{y} = \boldsymbol{y} - q\boldsymbol{t}. \tag{16}$$

Then the remaining column vectors of $\boldsymbol{W}$ and $\boldsymbol{P}$ are calculated iteratively based on the deflated $\boldsymbol{X}_g, \boldsymbol{X}_{iso}$, $\boldsymbol{X}$, $\boldsymbol{y}_g$ and $\boldsymbol{y}$. Specifically, we calculate $\boldsymbol{w}_{iso}$, $\boldsymbol{p}$ and q for the next dimension of the LV space by looping Eq (1)-(11). Finally, we obtain the n_k column vectors of the matrices $\boldsymbol{W}$ and $\boldsymbol{P}$, and the n_k elements of the vector $\boldsymbol{q}$ by iterating the above procedure n_k times.

Computing prediction scores of isoforms. The regression coefficient vector $\boldsymbol{b}$ can be calculated as:

$$\boldsymbol{b} = \boldsymbol{W}(\boldsymbol{P}^T\boldsymbol{W})^{-1}\boldsymbol{q} \tag{17}$$

Finally, the prediction scores of isoforms can be calculated below:

$$\boldsymbol{y}_{pred} = \boldsymbol{X}_{iso}\boldsymbol{b} \tag{18}$$

Supplementary Note S2. The procedure of model searching in the feature selection framework of IsoFrog [4, 5, 6].

Algorithm 1 Model searching

Objective: Form candidate feature subsets $\boldsymbol{V}'$ for jumping and determine whether to perform it.

for $n \leq N$ **do**
    Sampling $\boldsymbol{Q}'$ from $\boldsymbol{N}(\boldsymbol{Q}_0, \boldsymbol{\alpha}\boldsymbol{Q}_0)$;
    if $\boldsymbol{Q}' = \boldsymbol{Q}_0$ **then**
        $\boldsymbol{V}' = \boldsymbol{V}_0$;
    end if
    if $\boldsymbol{Q}' < \boldsymbol{Q}_0$ **then**
        Input $\boldsymbol{V}_0$ in $MdiPLS$ to obtain the regression coefficients $\boldsymbol{b}_0$ for $\boldsymbol{V}_0$;
        Sort variables in $\boldsymbol{V}_0$ in descending order according to $\boldsymbol{abs}(\boldsymbol{b}_0)$;
        $\boldsymbol{V}' =$ the top $\boldsymbol{Q}'$ features in $\boldsymbol{V}_0$;
    end if
    if $\boldsymbol{Q}' > \boldsymbol{Q}_0$ **then**
        Sampling $\boldsymbol{\mu}(\boldsymbol{Q}' - \boldsymbol{Q})$ variables from set $\boldsymbol{V} - \boldsymbol{V}_0$ and combining these sampled variables with $\boldsymbol{V}_0$ to obtain $\boldsymbol{V}_c$
        Input $\boldsymbol{V}_c$ in $MdiPLS$ to obtain the regression coefficients $\boldsymbol{b}_c$ for $\boldsymbol{V}_c$;
        Sort variables in $\boldsymbol{V}_c$ in descending order according to $\boldsymbol{abs}(\boldsymbol{b}_c)$;
        $\boldsymbol{V}' =$ the top $\boldsymbol{Q}'$ features in $\boldsymbol{V}_c$;
    end if
    $\boldsymbol{V}_0 \rightarrow MdiPLS \xrightarrow{2-fold\ CV} \boldsymbol{AUC}_0$;
    $\boldsymbol{V}' \rightarrow MdiPLS \xrightarrow{2-fold\ CV} \boldsymbol{AUC}'$;
    if $\boldsymbol{AUC}' \geq \boldsymbol{AUC}_0$ **then**
        $\boldsymbol{V}_0 = \boldsymbol{V}'$;
    end if
    if $\boldsymbol{AUC}' < \boldsymbol{AUC}_0$ **then**
        $\boldsymbol{prob}_{accept} = \omega \times \frac{\boldsymbol{AUC}' + 0.001}{\boldsymbol{AUC}_0 + 0.001}$;
        Sampling $\boldsymbol{p}$ from $\boldsymbol{U}(0, 1)$;
        if $\boldsymbol{p} \leq \boldsymbol{prob}_{accept}$ **then**
            $\boldsymbol{V}_0 = \boldsymbol{V}'$
        end if
    end if
end for

Supplementary Note S3. The procedure of Sequential Feature Selection.

Once we have the importance of each feature, we perform feature selection using a procedure called Sequential Feature Selection (SFS). The optimal number of features that should be input into the predictor is determined in this procedure. Our SFS is detailed below:

(1) List the selection probabilities of all the features and sort them in descending order. If there are repetitive values in it, only one of them is retained.

(2) Equally partition the list into 20 sub-lists with its 20-quantiles. The 20-quantiles are extracted to form a new threshold list. 0 is also added to the list. Besides, we take the quintiles of the upper two sub-lists that contain the maximum selection probabilities into the threshold list. The thresholds in the list are then arranged in ascending order.

(3) For each threshold in the list, the features with selection probabilities exceeding the threshold are extracted to form a feature subset that corresponds to the threshold.

(4) Input the universal set of all the features into MdiPLS to obtain an AUC with the 3-fold cross-validation (CV).

(5) For each threshold in the list, we input the corresponding feature subset to MdiPLS and evaluate its performance with the AUC obtained from the 3-fold CV.

(6) The set of features that maximize the performance of MdiPLS is the set of features we then work with. Note that the entire procedure works with the same values for the hyperparameters.

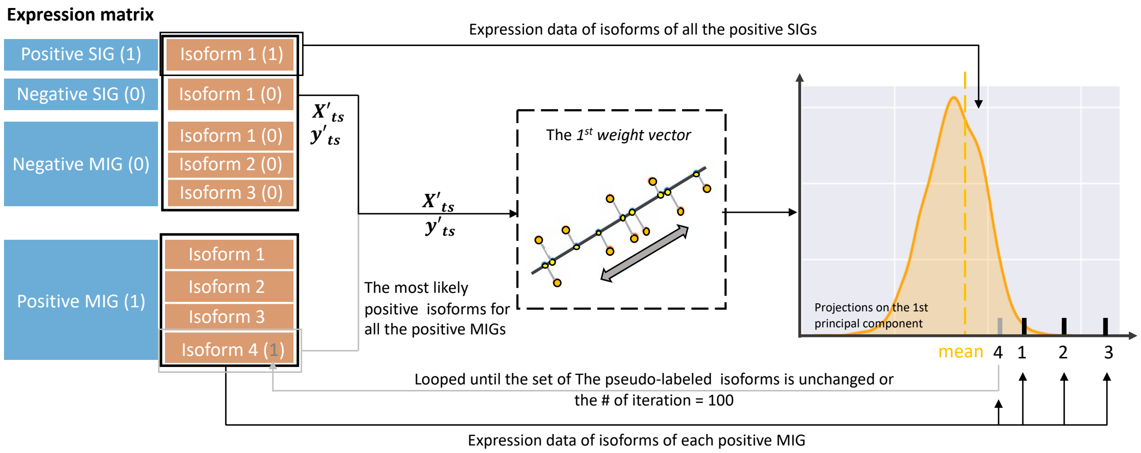

Figure S1. Schematic illustration of the MIL process in MdiPLS

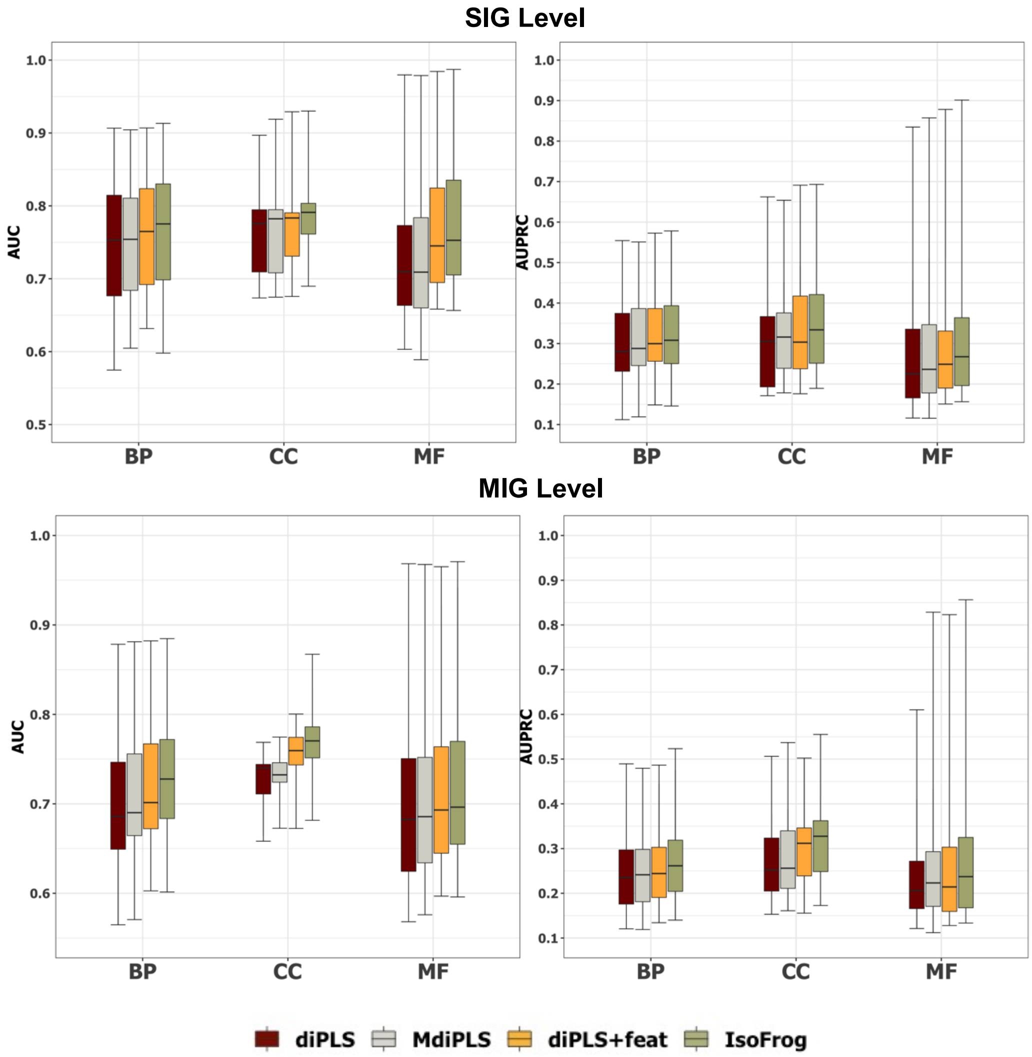

Figure S2. Comparison of prediction performance between diPLS, MdiPLS, diPLS-feat, and IsoFrog on different GO term categories. Specifically, MdiPLS integrates diPLS and the MIL update process in IsoFrog, while diPLS-feat integrates diPLS and the feature selection framework in IsoFrog

Table S1. Summary description of the datasets used in our study.

Datasets	#Genes	#isoforms	#Features	Refs
Dataset A	19303	39375	1735	Chen et al., 2019 [7]
Dataset B	19201	24274	365	Eksi et al., 2013 [8]
Dataset C	19217	34340	456	Li et al., 2014 [9]

* For Dataset A, B and C, the number of features is equal to the number of RNA-seq samples in which isoform expression is measured.

Table S2. Parameters of IsoFrog used in the experiments.

N	Q	α	μ	ω
2,000	200	0.3	3	0.02

Table S3. Results of the ablation experiment on Dataset A.

	diPLS	MdiPLS	diPLS+feat	IsoFrog
**AUC**	0.715	0.723	0.738	0.745
**AUPRC**	0.242	0.248	0.253	0.263

References:

1. Abdi, H. (2010). Partial least squares regression and projection on latent structure regression (PLS regression). Wiley Interdisciplinary Reviews: Computational Statistics, 2(1), 97–106.

2. Nikzad-Langerodi, R. et al. (2018). Domain-invariant partial-least-squares regression. Anal Chem, 90(11), 6693–6701.

3. Zhang, Y. et al. (2004). Scoring function for automated assessment of protein structure template quality. Proteins, 57, 702-710.

4. Lopes, H. F. (2006). A note on reversible jump 9arkov chain monte carlo. Graduate School of Business, The University of Chicago.

5. Green, P. J. (1995). Reversible jump 9arkov chain monte carlo computation and

10arkov10n model determination. Biometrika, 82(4), 711–732.

6. Li, H.-D. et al. (2012). Random frog: an efficient reversible jump 10arkov chain monte carlo-like approach for variable selection with applications to gene selection and disease classification. Analytica Chimica Acta, 740, 20–26.

7. Chen, H. et al. (2019). DIFFUSE: predicting isoform functions from sequences and expression profiles via deep learning. Bioinformatics, 35(14), i284–i294.

8. Eksi, R. et al. (2013). Systematically differentiating functions for alternatively spliced isoforms through integrating RNA-seq data. PloS Computational Biology, 9(11), e1003314.

9. Li, W. et al. (2014). High-resolution functional annotation of human transcriptome: predicting isoform functions by a novel multiple instance-based label propagation method. Nucleic Acids Research, 42(6), e39–e39.
